# Autologous adipose-derived stromal vascular fraction and scarred vocal folds: first clinical case report

**DOI:** 10.1186/s13287-018-0842-0

**Published:** 2018-07-27

**Authors:** Alexia Mattei, Jérémy Magalon, Baptiste Bertrand, Fanny Grimaud, Joana Revis, Mélanie Velier, Julie Veran, Patrick Dessi, Florence Sabatier, Antoine Giovanni

**Affiliations:** 1 Aix Marseille Univ, 13000 Marseille, France; 2 Department of Oto-Rhino-Laryngology and Head and Neck Surgery, Assistance Publique-Hôpitaux de Marseille, La Conception University Hospital, 13385 Cedex Marseille, France; 3 Aix Marseille Univ, C2VN, INSERM UMR 1263, Faculté de Pharmacie de Marseille, 27, Boulevard Jean Moulin, 13385 Marseille Cedex 5, France; 4 Cell therapy department, INSERM CBT-1409, Assistance Publique - Hôpitaux de Marseille, La Conception University Hospital, 13385 Cedex Marseille, France; 5 Department of Plastic and Reconstructive Surgery, Assistance Publique-Hôpitaux de Marseille, La Conception University Hospital, 13385 Cedex Marseille, France; 6 Aix Marseille Univ, CNRS, Laboratoire Parole et Langage, 5 Avenue Pasteur, 13100 Aix-en-Provence, France; 7 Aix-Marseille Univ, Anthropology ADES UMR 7268 AMU EFS CNRS, 13385 Cedex Marseille, France

**Keywords:** Stromal vascular fraction, Adipose tissue, Cell therapy, Vocal fold, Fibrosis, Scar, Dysphonia

## Abstract

Patients with scarred vocal folds (congenitally or following phonosurgery) are currently difficult to treat and present a dysphonia, often disabling in daily life. Several therapies are available on the market but the results of these are often disappointing. The autologous adipose-derived stromal vascular fraction (ADSVF) is recognized as an easily accessible source of cells with angiogenic, anti-inflammatory, immunomodulatory, and regenerative properties. We present here the case of a 43-year-old woman who had a severe dysphonia associated with scarred vocal folds after a phonosurgery and was resistant to conventional medical and surgical treatments. She received a local administration of autologous ADSVF. The protocol involved, on the same day, adipose tissue extraction, ADSVF preparation, and then local injection (0.45ml of ADSVF in each vocal fold, for a total of 12.2 million ADSVF viable cells). No serious adverse events have been described. One year following the surgery, the laryngoscopic aspect and the majority of voice parameters had improved, in particular the Voice Handicap Index decreasing from 75 to 9. The perceptual analysis found the voice to be less hoarse and more stable, without breathiness. The case of this patient highlights the therapeutic potential of ADSVF for such applications (trial registration, ClinicalTrials.gov NCT02622464; registered 4 December 2015).

## Introduction

Vocal fold (VF) microstructure [1, 2] is complex, particularly due to its foliated organization. The proportion and organization of the extracellular matrix components determine the mechanical properties of the VF. Following laryngeal microsurgery, VF scarring is sometimes observed, due to partial disappearance of the lamina propria, with the superficial and/or intermediate layer replaced by fibrous tissue, preventing mechanical uncoupling of the epithelium and muscle and thereby inducing vibration disorder [1]. Scar tissue may also be found congenitally, without iatrogenic etiology (e.g., sulcus vocalis).

VF scarring, depending on severity and extent, can result in a range of symptoms such as hoarseness, breathy voice, increased effort to speak, and voice fatigue. The inability to phonate normally causes both physical and psychological disability, especially for professional communicators. Several therapies are currently available but these are often disappointing as the great complexity of VF microstructure hinders the development of effective therapy. Thus, identification of innovative strategies able to improve vibrational mechanical properties of VF remains an important clinical challenge.

The autologous adipose-derived stromal vascular fraction (ADSVF) is recognized as an easily accessible source of cells displaying angiogenic, anti-inflammatory, immunomodulatory, and regenerative properties [3]. Recent experimental and clinical reports also support the anti-fibrotic potential of ADVSF, mainly attributed to the mesenchymal stem/stromal cell subset. Although ADSVF-based therapy remains totally unexplored in the field of laryngology, local injection of ADSVF could beneficially improve scarred VF. We obtained approval from the French National Agency for Medicine and Health Product Safety and the French Ethics Committee to deliver ADSVF in eight patients presenting with scarred VF that were refractory to conventional medical and surgical treatments (NCT02622464). Here, we present the first case of a patient who has had her 12-month follow-up examination.

## Methods

### Patient

A 43 year-old woman was included in the prospective phase I clinical trial CELLCORDES (EudraCT number 2015–000238-31) registered at clinicaltrials.gov, after providing written informed consent.

Her medical history included a thyroidectomy for goiter and hysterectomy for endometriosis. She presented a severe dysphonia related to scarred VF following a phonosurgery. Five years earlier, she had two laryngeal surgeries: i) suspension laryngoscopy for resection of Reinke edema, in which a right ventricular lesion was discovered and biopsied; ii) cervicotomy with lateral thyrotomy for resection of this lesion, whose definitive histology was in favor of a chondroma. One year later, the patient underwent another suspension laryngoscopy for a granuloma excision in the anterior third of the left VF. The laryngeal pathologies treated did not recur but the persistence of a marked dysphonia 4 years following the last surgery (despite regular speech therapy) justified a new consultation. The patient met the inclusion and exclusion criteria of the clinical trial (listed in Table 1) and thus was eligible to receive a local injection of autologous ADSVF.

**Table 1:** Inclusion and non inclusion criteria for the CELLCORDES clinical trial

Inclusion criteria	
- Affiliation to social security - Signed informed consent - Voice Handicap Index > 60/120 - Scarred vocal folds, congenital (sulcus) or after phonosurgery - Scarred middle third in stroboscopy - One year delay after initial surgery - Patients aged between 18 and 65 years - Good general condition - Negative pregnancy test and contraception for women of child-bearing age	
Non inclusion criteria	
Specific	Non-specific
- Refusal of speech therapy - History of malignant lesion or severe dysplasia of the scarred vocal fold - Contraindication to anesthesia - Anti-coagulant treatment - Coagulation disorders (prothrombin time < 65%, kaolin-activated partial thromboplastin time > 1,2) - Active infectious diseases - Positive serology for HIV, HBV, HCV, HTLV or syphilis - Necessity of intraoperative prophylactic antibiotics	- Taking an investigational medicinal product in the last 3 months - Refusal or inability to comply with study procedures - Pregnant and lactating women - Patients under curatorship or tutorship - Persons residing in a public health or social institution - Minors - Persons not covered by a social security scheme - Persons deprived of liberty or detainees - Persons in emergency situations

In videolaryngostroboscopy, a scarred aspect of the VF was observed, particularly an absence of vibration of their middle third. The vocal assessment enabled us to objectify this dysphonia (Table 2), with the speech therapist reporting a hoarse, unstable, and slightly breathy voice, with inability to reach high notes. Additional movie and audio files show this in more detail (Additional files 1, 2 and 3).

**Table 2:** Vocal assessments performed preoperatively and 12 months after injection of adipose-derived stromal vascular fraction

		Preoperative vocal assessment	12-month vocal assessment
Self-assessment	VHI 30 (/ 120)	75	9
Perceptual analysis	GRB of the Hirano scale	G2R2B0	G1R1B0
Acoustic analysis	Fundamental frequency (Hz)	180	203
	Vocal range (Hz)	381	921
	Jitter (%)	4.6	0.2
	Signal to noise ratio (dB)	9.5	17.7
Aerodynamic analysis	MPT (s)	13.2	7.8
	Oral air flow (cm^3^/s)	203	198
	ESP at the phonatory threshold (hPa)	7.9	7.3

*VHI* Voice Handicap Index, *MPT* maximum phonation time, *ESP* estimated subglottic pressure

### Surgical procedure

**Fig. 1:**
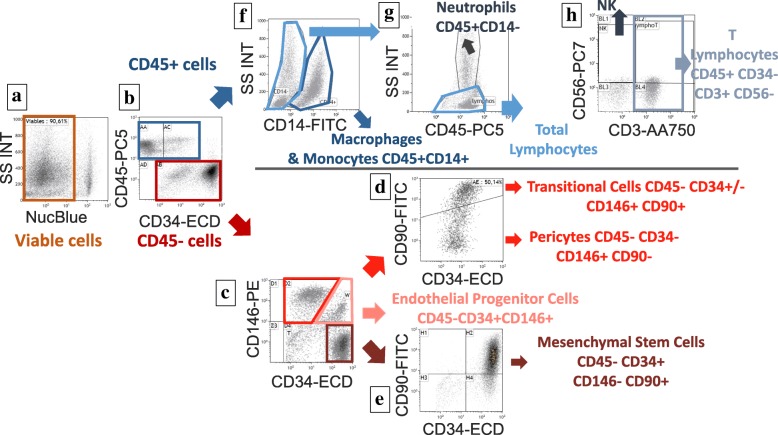
Flow cytometry analysis of SVF cell subsets representing the gating strategy divided among CD45+ (hematopoietic) and CD45- (regenerative) populations. (**a**) Selection of viable cells based on the negative expression of NucBlue and size scatter parameter (**b**) Viable cells are divided into CD45+ cells (hematopoietic) and CD45- cells (regenerative) (**c**) Among regenerative cells, three populations are identified based on the expression of CD146 and CD34: CD146high/CD34high are endothelial cells; CD146-/CD34high are stromal cells; CD146 high/CD34med population gathering pericytes and transitional cells (**d**) Pericytes and transitional cells are divided based on the expression of CD90 (+ for transitional and – for pericytes) (**e**) Confirmation of stromal cells identification based on the expression of CD90 (+ for stromal cells) (**f**) Among hematopoietic cells, macrophages are identified based on the expression of CD14 and size scatter parameter (**g**) Among CD14-/CD45+ cells, granulocytes and lymphocytes are separated based on the size scatter parameter (**h**) Expression of CD56 and CD3 allows to make characterization of NK and T lymphocytes subsets

The surgical procedure necessitated two consecutive surgeries performed on the same day. The first surgery consisted of an abdominal lipoaspiration, for ADSVF manufacturing, under local anesthesia with sedation. Harvesting of adipose tissue was performed with a 10 mL syringe in a closed circuit using a 3 mm Khouri cannula and a 500 mL collection bag; 340 ml of lipoaspirate was collected, transported to our registered Cell Therapy Unit, and transferred into the Celution 800/CRS system (Cytori therapeuthics Inc., San Diego, CA, USA). Collected lipoaspirate was washed and enzymatically digested using GMP grade reagents. Cells were concentrated, washed, aseptically recovered, and re-suspended in 5 mL Lactate Ringer’s solution. One milliliter was sampled for injection and the remaining cells were used for sterility testing and biological characterization and frozen in a biobank for research purposes. Total viable nucleated cell recovery and cell viability were determined using the Nucleocounter NC100 instrument (ChemoMetec, Denmark). Cellular components within isolated ADSVF were identified by flow cytometry analysis (Beckman Navios instrument) using a panel of cell surface makers in agreement with the International Federation for Adipose Therapeutics and Science (IFATS) and the International Society for Cellular Therapy (ISCT) recommendations [4] including NucBlue as viability marker and the fluorochrome-conjugated antibodies CD14-FITC, CD90-FITC, CD146-PE, CD34-ECD, CD45-PC5, CD56-PC7, and CD3-APC-A750 or their isotype control to determine the non-specific fluorescence (Fig. 1).

The second surgery consisted of the re-injection of ADSVF under general anesthesia. The ADSVF suspension was injected using a 14 G needle into the middle third of both scarred VF (0.45 mL per VF).

The patient was able to return home the next day, with antibiotic therapy of amoxicillin and analgesia by paracetamol. Perioperative speech therapy was prescribed.

### Assessment

The primary safety endpoint was the assessment of potential adverse events at day 7 and months 1, 6, and 12. The secondary endpoint assessed efficacy, defined as the improvement of vocal assessment, self-evaluation, and vibration in videolaryngostroboscopy at months 1, 6, and 12.

Videolaryngostroboscopy allows the assessment of glottal closure, regularity, mucosal wave, and symmetry. Self-evaluation was made using the Voice Handicap Index (VHI), with lower scores indicating a better voice perception (a shift in the total score of 18 points or greater is required to be clinically significant [5]). The severity of dysphonia was quantified with the simplified GRB scale derived from the GRBAS scale proposed by Hirano [6] (G for global, R for roughness, B for breathiness). Jitter refers to a short-term (cycle-to-cycle) perturbation in the fundamental voice frequency (the lower the better). The signal to noise ratio quantifies the aperiodic portion of the voice signal (the higher the signal the better). Maximum phonation time (MPT) and oral airflow (OAF) quantify the glottal air leakage: if the leakage is significant, MPT should be short and OAF high. The estimated subglottic pressure (ESP) at the phonatory threshold is the minimum subglottic pressure needed to initiate and sustain VF vibration [7].

## Results

ADSVF characteristics are summarized in Table 3.

**Table 3:** Characteristics of adipose-derived stromal vascular fraction

Volume of adipose tissue harvested (cc)	340
Volume of ADSVF injected (ml)	2 × 0.45
Number of VNC obtained, before quality control (millions)	135
Recovery rate (VNC/cc of adipose tissue)	397,000
Number of VNC cells injected, after quality control (millions)	12.2
Viability (%)	86
Leukocytes (%)	35
*Macrophages/monocytes (%)*	17.6
*Lymphocytes (%)*	12.8
*Neutrophils* *(%)*	4.6
Transitional cells (%)	3.8
Endothelial progenitor cells (%)	2.7
Pericytes (%)	3.8
Mesenchymal stem cells = adipose-derived stem cells (%)	54.7

*ADSVF* adipose-derived stromal vascular fraction, *VNC* viable nucleated cells

The only adverse events reported were the existence of hematomas in the areas of liposuction (resorption in 3 weeks) and mandibular pain for 1 week (related to the installation required for laryngoscopy). No severe adverse event linked to cellular product administration was reported. Concerning efficacy, Table 2 indicates the results of the final evaluation at 12 months (additional movie and audio files show this in more detail (Additional files 4, 5 and 6)). The perceptual analysis found the voice to be less hoarse and more stable, without breathiness. The videolaryngostroboscopy found a significant improvement in the vibration of the middle third of the two VF. The patient reported being better heard by her entourage thanks to a “clearer” voice.


Additional file 4:Videolaryngostroboscopy one year after stromal vascular fraction injection: the vibration of the middle third of VF is improved. (MP4 9561 kb)


## Discussion

The vocal impact of scarred VF is disabling, while current treatment options are limited. The use of ADSVF-based cell therapy is particularly promising, since its effectiveness has already been proven in several pathologies [3, 8–11], but it had so far never been tested in humans for this current pathology. Adipose-derived stem cells (ADSC), a purified and ex vivo expanded multipotent mesenchymal/stromal cell population from adipose tissue, have shown, in animal models of scarred VF, their ability to decrease inflammation and fibrosis and to improve the viscoelastic properties of the mucosa [12, 13]. To our knowledge this is the first published case of uncultured and minimally processed ADSVF injection at the laryngeal level in humans. The ADSVF presents a very fluid consistency, allowing its injection into the Reinke space, thus fighting against the adhesion between the epithelium and the underlying tissues. We have deliberately chosen not to associate an excision of the fibrous tissues, in order to avoid increased atrophy of the VF. Findings suggest a clear improvement in the majority of the parameters of the voice assessment, in particular the VHI, illustrating the patient’s own perception, with no severe adverse or unexpected events.

Seven other patients were included in this trial. Their follow-up is not finished yet but it seems that three of them also have very good results (especially on the VHI). None of them had any severe adverse effects or worsening.

Previous preclinical and clinical studies suggest that ADSVF exerts anti-fibrotic effects. In a scleroderma-like skin sclerosis in nude mice, Serratrice et al. [14] showed that ADSVF significantly reversed dermal and epidermal sclerosis and was associated with a significant increase of the local vascularization. Domergue et al. [15] compared the whole ADSVF cell product and cultured ADSC from adipose tissue in a humanized skin graft model of hypertrophic scar in nude mice. They reported that both cell-based therapeutic strategies were able to significantly reduce the clinical and histological parameters of hypertrophic scar. Through the secretion of adrenomodullin and hepatocyte growth factor, ADSC reduced expression of TGFβ1 and its target genes (collagen I, collagen III, α-SMA). Although only significant for TGF-β1, they observed the down-regulation of all genes at day 8 following ADSVF injection, but not following ADSC injection. On the other hand, ADSC induced a highly significant increase of TGFβ3 expression, with a change in the TGFβ1/TGFβ3 ratio in favor of an anti-fibrotic effect. ADSC also tended to increase MMP-1 and MMP-3 and significantly up-regulated the MMP-2 and MMP-2/TIMP-2 ratio, while ADSVF cells did not.

Thus, the beneficial effect in the reported case could be attributed to the presence of ADSC within the ADSVF, representing 54.7% of the 12.2 million viable injected cells.

These therapies are regulated through regulation number 1394/2007 of the European Parliament and Council, describing a new category of health products named “Advanced Therapy Medicinal Products” (ATMPs). Production of ATMPs should comply with good manufacturing practice (GMP) of pharmaceutical industries, meaning an increase in the production costs, which may not be sustainable for public institutions. However, while expanding ADSC takes 2 to 3 weeks, ADSVF can be manufactured within a few hours, allowing lipoaspiration and re-injection on the same day. This point should be considered from a cost-effectiveness point of view.

This report encourages the inclusion and monitoring of a greater number of patients to document the safety and effectiveness of this therapy. Next, efficacy trials versus placebo should be considered to confirm the expected effectiveness. Indeed, an isolated or added volumizing effect is not excluded and a partial placebo effect is possible in these patients, who have high hopes for such therapies as they can be perceived to be futuristic. However, this case holds promise for future cell therapy in laryngeal pathologies such as scarred VF where available treatments are lacking. Among the panel of cell-based therapy products currently being developed, the ADSVF may have significant advantages because of its relative simplicity of production and use.

## Additional files

Additional file 1: Videolaryngostroboscopy before stromal vascular fraction injection: scarred aspect of the vocal folds with an absence of vibration in their middle third. (MP4 1489 kb)

Additional file 2: Voice recording during reading before stromal vascular fraction injection: hoarse, unstable, and slightly breathy voice. (WAV 2796 kb)

Additional file 3: Voice recording during a sustained vowel /a/ before stromal vascular fraction injection: hoarse, unstable, and slightly breathy voice. (WAV 1880 kb)

Additional file 5: Voice recording during reading one year after stromal vascular fraction injection: the voice is less hoarse and more stable, without breathiness. (WAV 1335 kb)

Additional file 6: Voice recording during a sustained vowel /a/ one year after stromal vascular fraction injection: the voice is less hoarse and more stable, without breathiness. (WAV 667 kb)

## Abbreviations

ADSC: Adipose-derived stem cells
ADSVF: Adipose-derived stromal vascular fraction
ESP: Estimated subglottic pressure
GMP: Good manufacturing practice
MPT: Maximum phonation time
OAF: Oral air flow
VF: Vocal fold(s)
VHI: Voice handicap index
VNC: Viable nucleated cells
